# Design of Iodonium Salts for UV or Near-UV LEDs for Photoacid Generator and Polymerization Purposes

**DOI:** 10.3390/molecules25010149

**Published:** 2019-12-30

**Authors:** Ségolène Villotte, Didier Gigmes, Frédéric Dumur, Jacques Lalevée

**Affiliations:** 1 Aix Marseille Univ, CNRS, ICR UMR 7273, 13397 Marseille, France; segolene.villotte@etu.univ-amu.fr (S.V.); didier.gigmes@univ-amu.fr (D.G.); 2 Université de Haute-Alsace, CNRS, IS2M UMR 7361, F-68100 Mulhouse, France; 3 Université de Strasbourg, 67000 Strasbourg, France

**Keywords:** iodonium salt, photoacid, photopolymerization, cationic initiator, LED

## Abstract

Iodonium salts are well established photoacid generators, cationic photoinitiators, as well as additives commonly used in photoredox catalytic cycles. However, as a strong limitation, iodonium salts are characterized by low light absorption properties for λ > 300 nm so that these latter cannot be activated with cheap, safe, and eco-friendly near UV or even visible light emitting diodes (LEDs). To overcome this drawback, the covalent linkage of an iodonium salt to a chromophore absorbing at longer wavelength is actively researched. With aim at red-shifting the absorption spectrum of the iodonium salt, the synthesis of new compounds combining within a unique chemical structure both the chromophore (here the naphthalimide scaffold) and the iodonium salt is presented. By mean of this strategy, a polymerization could be initiated at 365 nm with the modified iodonium salts whereas no polymerization could be induced with the benchmark iodonium salt i.e., Speedcure 938 at this specific wavelength. To examine the effect of the counter-anion on the photoinitiating ability of these different salts, five different counter-anions were used. Comparison between the different anions revealed the *bis*(trifluoromethane)sulfonimide salt to exhibit the best photoinitiating ability in both the free radical polymerization of acrylates and the cationic polymerization of epoxides. To support the experimental results, molecular orbital calculations have been carried out. By theoretical calculations, the initiating species resulting from the photocleavage of the iodonium salts could be determined. The cleavage selectivity and the photochemical reactivity of the new iodoniums are also discussed.

## 1. Introduction

The light induced cationic polymerization (CP) is already characterized by a widespread use in the radiation curing area (see e.g., in [1,2,3,4,5,6,7,8,9,10,11,12,13,14]). Notably, photoinduced cationic polymerization is widely used in coatings [15], adhesives [16], microelectronics [17], graphic arts [18], dentistry [19], and 3D-printing [20,21] and these different applications can be cited as the most relevant ones.

Among cationic initiators, diaryliodonium, triarylsulfonium salts [1,2,3,4,5,6,7,8,9,10,11,12,13,14,22,23,24,25] and others [26,27,28,29,30,31,32,33,34] have been extensively studied in the literature. The chemistry of iodonium salts is notably well-documented in the literature [1,2,3,4,5,6,7,8,9,10,11,12,13,14,22,23,24,25]. Besides, the reported structures still suffer from poor light absorption properties for wavelengths higher than 300 nm, so that these different structures can only be activated with UV light [26,27,28,29,30,31,32,33,34]. Even if some of the onium salts were proposed for absorption at longer wavelengths (e.g., thianthrenium salts, thioxanthenium salts, modified iodonium or sulfonium salts, pyridinium salts) [35,36,37,38,39,40,41,42,43,44,45,46], the current works are more oriented to the use of external photosensitizers and/or free-radical sources to extend the spectral response of these different salts [1,2,3,4,5,6,7,8,9,10,11,12,13,14]. At present, only few works have been devoted to expanding the spectral range of iodonium salts by the covalent linkage of chromophores to the iodonium salts [47,48,49]. In fact, a careful survey of the literature reveals these chemical modifications of iodonium salts to be limited to the replacement of one the phenyl ring by a naphthalene or a biphenyl group what is definitely insufficient to drastically red-shift the absorption of the corresponding iodonium salts [47,48,49]. A breakthrough can be achieved by using chromophores other than phenyl or naphthalene so that the absorption spectra can be clearly displace towards the visible region, what is possible with naphthalimides. Choice of this chromophore was supported by previous works done by our groups evidencing nahphtalimide to be an excellent candidate for the design of type II photoinitiators [50,51,52,53,54,55,56,57,58,59,60].

**Figure 1:**
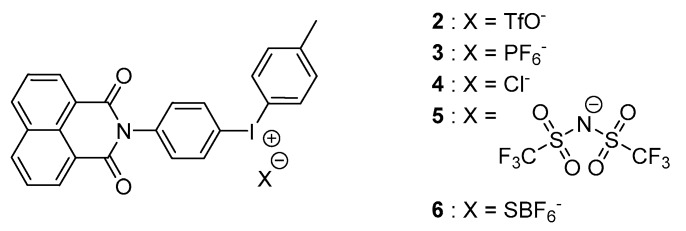
Chemical structures of the different iodonium salts **2**–**6**.

**Scheme 1:**
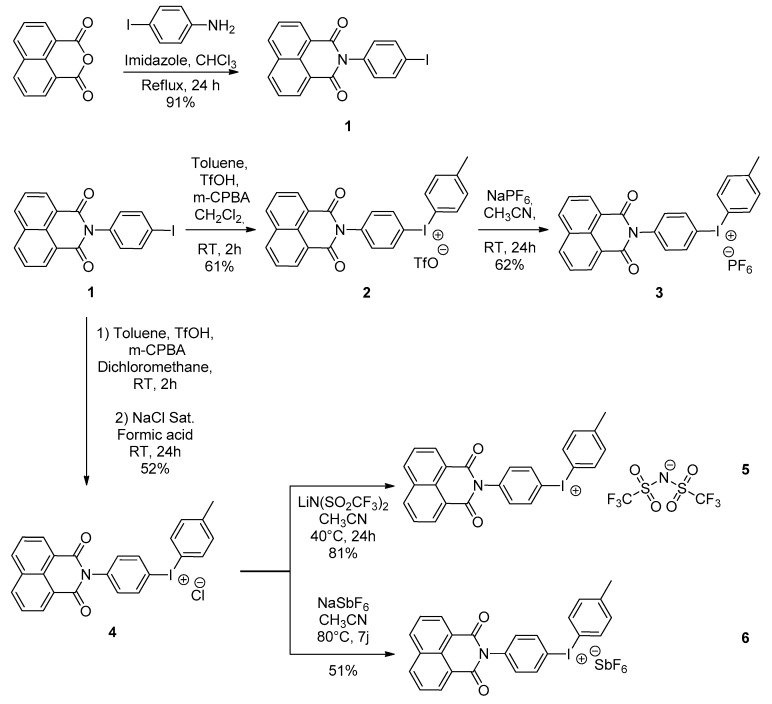
Synthetic pathways to iodonium salts **2**–**6**.

**Scheme 2:**
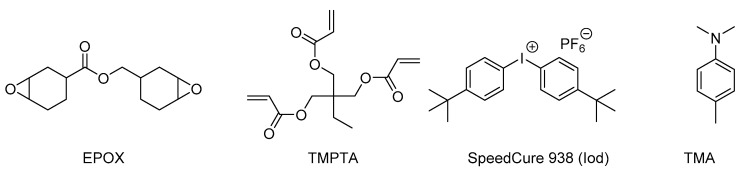
Chemical structures of Iodonium salt (Speedcure 938), amine (TMA) and benchmark monomers used.

In this context, naphthalimide was selected as a possible candidate to act as a covalently linked photosensitizer for diaryliodonium salts [7]. Therefore, in the present paper, a series of five new photosensitive iodonium salts (noted **2**–**6** in Figure 1; synthesis described in Scheme 1) differing by the counter-anions and resulting from a coupling between both a naphthalimide chromophore and an iodonium moiety is proposed to improve the light absorption properties in the 350–380 nm range. Interestingly, Speedcure 938 i.e., *bis*-(4-*tert*-butylphenyl)-iodonium hexafluorophosphate is totally ineffective to initiate a polymerization at such wavelength without taking recourse to a photosensitizer. The approach presented in this work is interesting to generate initiating species through an intramolecular process (here a monocomponent system) instead of an intermolecular reaction in a two-component photosensitizer/diaryliodonium system. Especially in this work, the higher efficiency of the monocomponent system over the bicomponent system making using of the benchmark Speedcure 938 is clearly evidenced. The polymerization of a benchmark epoxy monomer i.e., (3,4-epoxycyclohexane)methyl 3,4-epoxycyclo-hexylcarboxylate (EPOX) is monitored by time resolved infrared spectroscopy. The performance of the proposed iodonium salts is compared to a commercial reference, namely Speedcure 938 (See Scheme 2), exhibiting a significant absorption only in the 230–300 nm range. A comparison of the performances obtained while using different counter anions is also proposed. The photochemical cleavage processes are studied by electron spin resonance experiments and molecular orbital (MO) calculations.

## 2. Results

**Figure 2:**
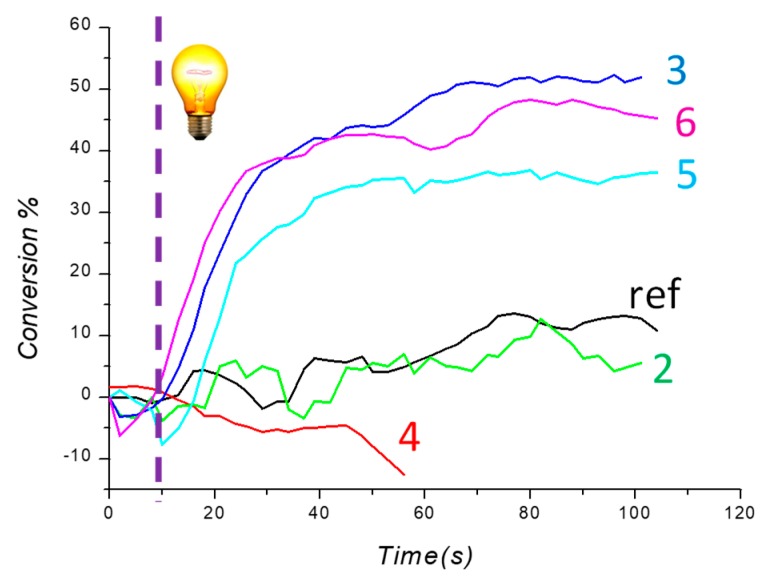
Photopolymerization profiles (epoxy function conversion vs. irradiation time) measured by RT-FTIR for the photoinduced polymerization of EPOX using the different iodoniums (**2**–**6**) 1% *w*/*w*; the polymerization profile obtained with the reference is noted ref; thin samples (25 μm) under air, upon LED@365 nm irradiation (the irradiation starts at t = 10 s, 500 mW/cm^2^).

**Figure 3:**
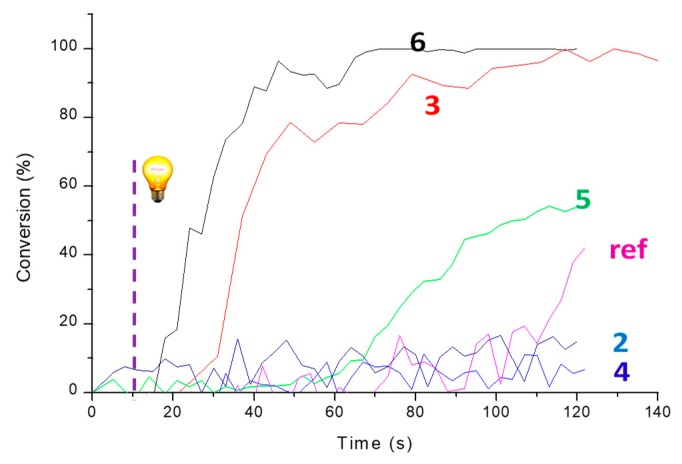
Photopolymerization profiles (epoxy function conversion vs. irradiation time) measured by RT-FTIR for the photoinduced polymerization using the different iodoniums (**2**–**6**) 1% *w*/*w*; the polymerization profile obtained with the reference is noted ref; thick samples (1.4 mm) under air, upon LED@365 nm irradiation (the irradiation starts at t = 10 s, 500 mW/cm^2^).

**Figure 4:**
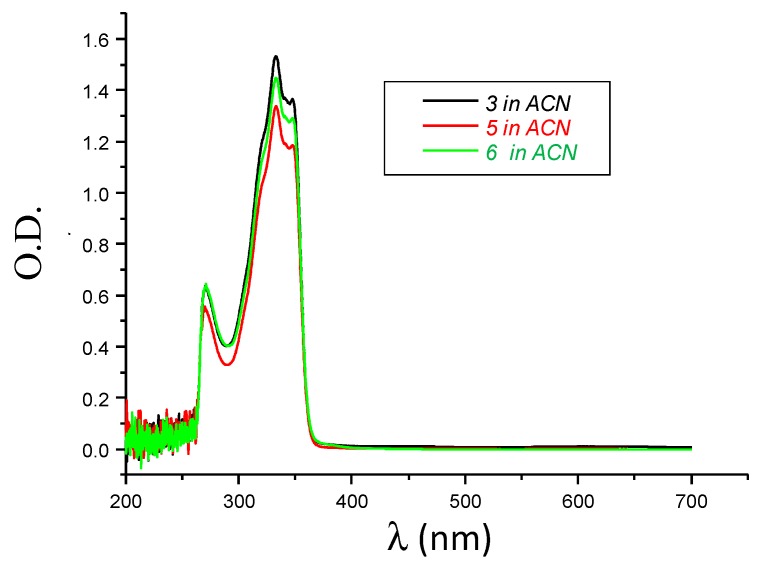
UV-vis absorption spectra for the most reactive iodonium salts (**3**, **5**, and **6**) in acetonitrile for a concentration of 1 × 10^−4^ M.

**Figure 5:**
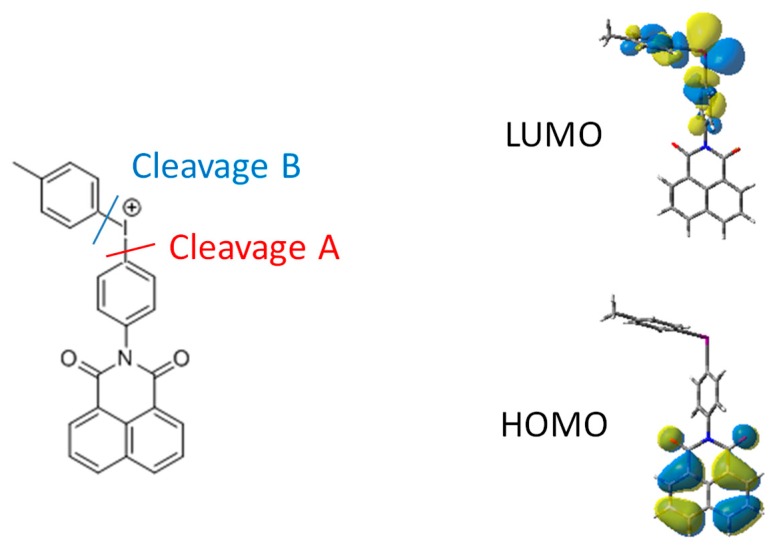
Investigated cleavage processes (Cleavage A and Cleavage B); highest occupied and lowest unoccupied molecular orbitals (HOMO and LUMO), respectively.

The different iodonium salts **2**–**6** were first investigated for their photoinitiating ability for the cationic polymerization of epoxides in thin (See Figure 2) and thick (See Figure 3) samples upon irradiation with a LED at 365 nm. Remarkably, it can be observed that **3**, **5**, and **6** lead to good polymerization performances that are clearly better than that of the reference Speedcure 938 for which no polymerization occurred. These results are in agreement with their significant light absorption properties in the 350–400 nm region (See Figure 4). On the opposite, as the reference Speedcure 938 does not absorb for λ > 300 nm [5] so that no polymerization is possible upon irradiation at 365 nm with this compound. Conversely, the maximum absorption wavelength of **3**, **5**, and **6** is close to 345 nm with a molar extinction coefficient higher than 10,000 M^−1^ cm^−1^ so that the absorption is sufficient to efficiently initiate a polymerization process. From the theoretical calculations (See Figure 5), it can be observed that the naphthalimide chromophore strongly participates to the highest occupied molecular orbital (HOMO), destabilizing the HOMO level and decreasing the HOMO–LUMO (lowest unoccupied molecular orbital) gap in the iodonium salts **2**–**6**. As a result of this, a bathochromic shift of the absorption spectra for **2**–**6** is observed compared to that of the reference Speedcure 938.

The light absorption properties for the different iodonium salts originating from the iodonium salt and not from the counter anions, the difference of reactivity between **2**–**6** can be ascribed to the counter anion effect. Indeed, it is well established that low nucleophilicity counter anions are more favorable (SbF_6_^−^; PF_6_^−^; *bis*(trifluoromethane)sulfonamide) by preserving the cationic propagating centers compared to higher nucleophilic counter anions (Cl^−^; TfO^−^) [1,3,5].

## 3. Discussion

It is well established that the iodonium salts can be cleaved (C–I bond) upon light irradiation leading to the formation of strong acids (r1) ([1,3,5] and references herein) in agreement with a photoacid behavior:

R_1_–I^+^–R_2_/A^−^ →→→ H^+^/A^−^ **(hν)** (r1)

This behavior is observed here with **3**, **5**, and **6**. Indeed, during the irradiation of the solutions of iodonium salts in presence of methyl red, appearance of the protonated form of this pH-indicator was clearly observed by UV-visible spectroscopy. This photoacid ability is also in full agreement with their initiating properties for the polymerization of epoxides [61].

To further investigate the cleavage process, molecular orbital calculations were carried out. From the two possible cleavages (noted A and B in Figure 5), the cleavage B is more favorable than the cleavage A due to a lower C–I bond dissociation energy (BDE): 49.7 vs. 57.4 kcal/mol for cleavage B and A, respectively. This latter result is also in agreement with the electron spin resonance experiments. Indeed, the ESR-ST spectrum obtained upon irradiation of **3** in the presence of PBN as a spin-trap agent in *tert*-butylbenzene showed the formation of an aryl (Ar^•^)/PBN radical adduct (hyperfine coupling constants hfcs: a_N_ = 14.3 G and a_H_ = 2.2 G; reference values in [62]).

The wavelength 405 nm is important in 3D printing and laser write experiments [63], however, the light absorption properties of the new proposed iodoniums is not high sufficient to initiate a polymerization process at this wavelength. Recently, the possibility to create charge transfer complexes (CTC) between electron rich amines (e.g., trimethylaniline (TMA) in Scheme 2) and electron poor iodonium salts has been reported in the literature (r2) [63]. These CTC can be photolyzed at 405 nm, leading to an efficient release of aryl radicals Ar^●^ (here Tol^●^) (r3) to initiate radical polymerization processes.

Amine + Iodonium → [Amine–Iodonium]_CTC_ (r2)

[Amine–Iodonium]_CTC_ →→→ Ar^●^  **(hν)** (r3)

**Figure 6:**
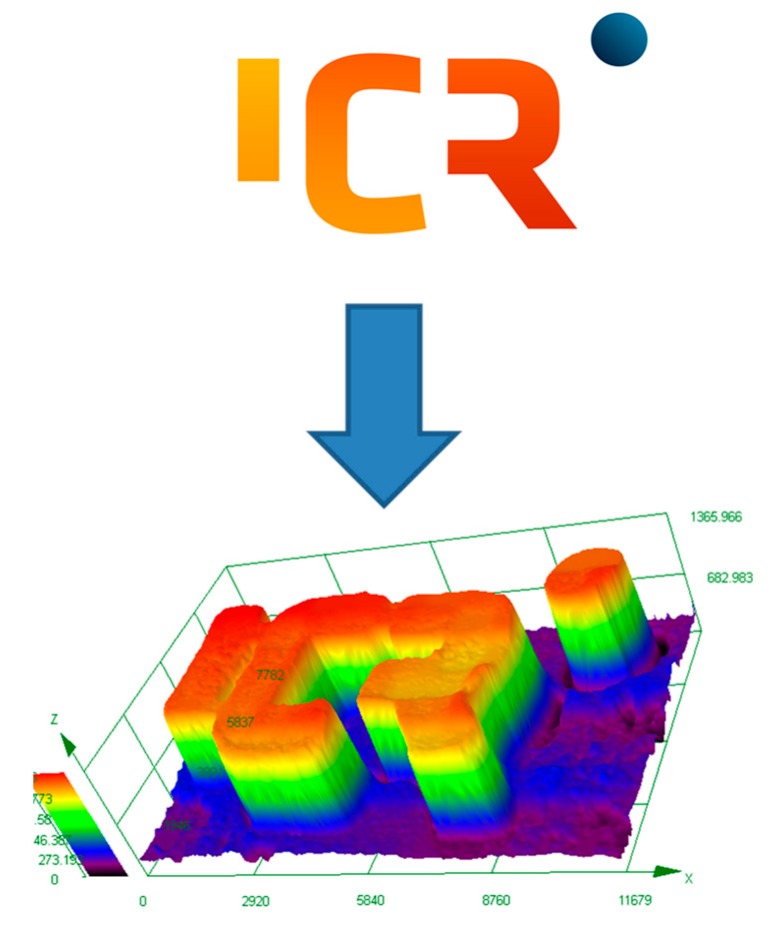
Numerical optical microscope observation of patterns “ICR” written from the polymerization of trimethylolpropane triacrylate (TMPTA) with the iodonium **5** in combination with trimethylaniline (TMA) (1%/1% *w*/*w*): 3D overall appearance. A writer using a laser diode at 405 nm (size of the spot around 50 μm) with the intensity of 100 mW was used for the spatially controlled irradiation.

This approach has notably been used here in laser write experiments @405 nm for the **5**/TMA system in trimethylolpropane triacrylate (TMPTA) used as a trifunctional acrylic monomer (Figure 6). No polymerization was observed while using **5** or TMA alone, showing the requirement of this combination through a CTC.

## 4. Materials and Methods 

### 4.1. Synthesis of the New Iodonium Salts

The general synthetic pathway for the iodonium salts **2**–**6** is depicted in Scheme 1. The procedure is described in detail below. The procedure has been inspired from [64].

All reagents and solvents were purchased from Aldrich (St. Louis, MO, USA) or Alfa Aesar (Haverhill, MA, USA) and used as received without further purification. Mass spectroscopy and NMR were performed by the Spectropole of Aix-Marseille University (Marseille, France). HRMS mass spectral analysis was performed with a SYNAPT G2 HDMS (Waters) mass spectrometer (*Waters* Corporation, Manchester, UK). ^1^H and ^13^C NMR spectra were recorded at room temperature in 5 mm o.d. tubes on a Bruker AC 400 spectrometer of the Spectropole (Billerica, MA, USA): ^1^H (400 MHz), ^19^F (376 MHz) and ^13^C (100 MHz). The ^1^H chemical shifts were referenced to the solvent peak (CD_3_)_2_CO (2.05 ppm), (CD_3_)_2_SO (2.49 ppm), CDCl_3_ (7.26 ppm) and the ^13^C chemical shifts, determined by ^13^C-APT experiment, were referenced to the solvent peak Acetone-D_6_ (29.80 ppm and 206.3 ppm), DMSO-D_6_ (39.52 ppm), CDCl_3_ (77.0 ppm).

*N-(p-iodophenyl)-1,8-naphthalimide* (**1**): A mixture of 1.8-naphthalic anhydride (2.10 g, 10.60 mmol, M = 198.17 g·mol^−1^), 4-iodoaniline (4.70 g, 21.46 mmol, 2.eq., M = 219.02 g·mol^−1^) and imidazole (14.17 g, 20.81 mmol, 20 eq., M = 68.08 g·mol^−1^) in chloroform (70 mL) was refluxed for 24 h. After cooling, the solvent was removed under reduced pressure. The crude product was taken up in absolute ethanol and the resulting suspension was sonicated for 15 min. The suspension was filtered off and washed with ethanol to afford the pure product as a grey solid (3.87 g, 9.69 mmol, 91% yield). ^1^H NMR (CDCl_3_) δ(ppm): 7.05–7.10 (m, 2H), 7.80 (t, 2H, *J* = 7.8 Hz), 7.85–7.90 (m, 2H), 8.28 (dd, 2H, *^3^J* = 8.3 Hz, ^4^*J* = 0.8 Hz), 8.64 (dd, 2H, ^3^*J* = 7.3 Hz, ^4^*J* = 0.9 Hz). ^13^C NMR (CDCl_3_) δ(ppm): 94.5 (Cq), 122.7 (Cq, 2C), 127.2 (CH, 2C), 128.6 (Cq), 130.8 (CH, 2C), 131.9 (CH, 2C), 131.8 (Cq), 134.6 (CH, 2C), 135.3 (Cq), 138.7 (CH, 2C), 164.2 (C=O, 2C). HRMS (ESI MS) *m*/*z*: theor: 399.9829 found: 399.9829 ((M + H)^+^ detected).

*(4-(1,3-Dioxo-1H-benzo[de]isoquinolin-2(3H)-yl)phenyl)(p-tolyl)iodonium trifluoro-methanesulfonate* (**2**): A mixture of *N*-(*para*-iodophenyl)-1,8-naphthalimide (0.67 g, 1.68 mmol, M = 399.18 g·mol^−1^), *m*-CPBA (77%) (0.39 g, 1.74 mmol, 1.04 eq., M = 172.57 g·mol^−1^), toluene (0.35 mL, 30.28 mg, 3.29 mmol, 1.95 eq., d = 0.865, M = 92.14 g·mol^−1^), and trifluoromethanesulfonic acid (0.35 mL, 59.36 mg, 3.96 mmol, 2.4 eq., d = 1.696, M = 150.08 g·mol^−1^) was stirred at room temperature for 2 h. The mixture was poured into diethyl ether and the resulting precipitate was filtered off and washed with diethyl ether to afford a pure product as a light yellow solid. (0.64 g, 1.02 mmol, 61% yield). ^1^H NMR (Acetone-d_6_) δ(ppm): 2.43 (s, 3H), 7.44 (d, 2H, *J* = 8.1 Hz), 7.71 (dt, 2H, ^3^*J* = 8.8 Hz, ^4^*J* = 2.4 Hz) 7.87 (dd, 2H, ^3^*J* = 8.2 Hz, ^4^*J* = 7.3 Hz), 8.31 (dt, ^3^*J* = 8.2 Hz, ^4^*J* = 2.4 Hz), 8.42–8.53 (m, 6H). ^19^F NMR (Acetone-d_6_) δ(ppm): −78.77 (s, 3F). ^13^C NMR (Acetone-d_6_) δ(ppm): 21.4 (CH_3_), 111.5 (Cq), 114.2 (Cq), 121.2 (q, CF_3_, ^1^*J*_C-F_ = 320 Hz) 123.6 (Cq, 2C), 128.0 (CH, 2C), 129.2 (Cq), 131.8 (CH, 2C), 132.8 (Cq), 133.9 (CH, 2C), 134.1 (CH, 2C), 135.5 (CH, 2C), 136.8 (CH, 2C), 136.9 (CH, 2C), 141.4 (Cq), 144.9 (Cq), 164.5 (C=O, 2C). HRMS (ESI MS) *m*/*z*: theor: 490.0299 found: 490.0302 ((M-TfO^−^)^+^ detected), theor 1129.0123, found 1129.0128 (((M^+^TfO^−^)M^+^) detected).

*(4-(1,3-Dioxo-1H-benzo[de]isoquinolin-2(3H)-yl)phenyl)(p-tolyl)iodonium* hexafluorophosphate (**3**): A mixture of (4-(1,3-dioxo-1*H*-benzo[*de*]isoquinolin-2(3*H*)-yl)phenyl)(*p*-tolyl)iodonium trifluoromethanesuflonate (205 mg, 0.33 mmol, M = 625.35 g·mol^−1^), and NaPF_6_ (331 mg, 0.197 mmol, 6 eq., M = 167.95 g·mol^−1^) in CH_3_CN (4.5 mL) was stirred for 24 h. Then, the solvent was removed under reduced pressure. Water was introduced and the resulting precipitate was filtered off and washed with water to afford a pure compound as a light yellow solid (130 mg, 0.20 mmol, 62% yield). ^1^H NMR (Acetone-d_6_) δ(ppm): 2.45 (s, 3H), 7.47 (d, 2H, *J* = 8.3 Hz), 7.73 (d, 2H, ^3^*J* = 8.7 Hz), 7.85–7.94 (m, 2H), 8.33 (d, 2H, ^3^*J* = 8.4 Hz), 8.47–8.55 (m, 6H). ^19^F (Acetone-d_6_) δ(ppm): −72.36 (d, 6F, ^1^*J*_P-F_ = 706.9 Hz). ^13^C NMR (Acetone-d_6_) δ(ppm): 21.4 (CH_3_), 111.2 (Cq), 114.0 (Cq), 123.7 (Cq, 2C), 128.0 (CH, 2C), 129.2 (Cq), 131.8 (CH, 2C), 132.9 (Cq), 134.1 (CH, 2C), 134.3 (CH, 2C), 135.5 (CH, 2C), 136.8 (CH, 2C), 136.9 (CH, 2C), 141.7 (Cq), 145.3 (Cq), 164.5 (C=O, 2C). HRMS (ESI MS) *m*/*z*: theor: 490.0299, found 490.0299 ((M-PF_6_^−^)^+^ detected), theor: 1125.0244 found: 1125.0243 (((M+PF_6_^−^)M^+^) detected).

*(4-(1,3-Dioxo-1H-benzo[de]isoquinolin-2(3H)-yl)phenyl)(p-tolyl)iodonium chloride* (**4**): A mixture of *N*-(*p*-iodophenyl)-1,8-naphthalimide (1.80 g, 4.51 mmol, M = 399.18 g·mol^−1^), *m*-CPBA (77%) (1.12g, 5.00 mmol, 1.12 eq., M = 172.57 g·mol^−1^), toluene (0.95 mL, 82.18 mg, 8.92 mmol, 1.98 eq., d = 0.865, M = 92.14 g·mol^−1^), and trifluoromethanesulfonic acid (0.35 mL, 59.36 mg, 3.96 mmol, 2.4 eq., d = 1.696, M = 150.08 g·mol^−1^) was stirred in dichloromethane (30 mL) at room temperature for 2 h. Then dichloromethane was removed under vacuum and the crude product was washed in 70 mL of diethyl ether. The solid was filtered off, dissolved in acetic acid. Brine is added until precipitation. The mixture is stirred at 80 °C during 24 h. The light-yellow solid is filtered, washed with 3 × 70 mL of demineralized water (1.24 g, 2.36 mmol, 52%). ^1^H NMR (DMSO-d_6_) δ(ppm): 2.36 (s, 3H), 7.35 (d, 2H, ^3^*J* = 8.2 Hz), 7.54 (d, 2H, ^3^*J* = 8.6 Hz), 7.91 (t, 2H, ^3^*J* = 8.2 Hz), 8.17 (d, 2H, ^3^*J* = 8.3), 8.31 (d, 2H, ^3^*J* = 8.6 Hz), 8.45–8.57 (m, 4H). ^13^C NMR (DMSO-d_6_) δ(ppm): 20.9 (CH_3_), 115.3 (Cq), 118.4 (Cq), 122.4 (Cq, 2C), 127.3 (CH, 2C), 127.8 (Cq), 130.8 (CH, 2C), 131.5 (Cq), 132.2 (CH, 2C), 132.3 (CH, 2C), 134.7 (CH, 2C), 135.1 (CH, 2C), 135.4 (CH, 2C), 139.0 (Cq), 142.2 (Cq), 163.5 (C=O, 2C). HRMS (ESI MS) *m*/*z*: theor: 490.0299, found 490.0299 ((M-Cl^−^)^+^ detected).

*(4-(1,3-Dioxo-1H-benzo[de]isoquinolin-2(3H)-yl)phenyl)(p-tolyl)iodonium bis((trifluoromethyl) sulfonyl)amide* (**5**): A mixture of (4-(1,3-dioxo-1*H*-benzo[*de*]isoquinolin-2(3*H*)-yl)phenyl)(*p*-tolyl)iodonium chloride (0.50 g, 0.95 mmol, M = 527.77 g·mol^−1^) and *bis*(trifluoromethane)sulfonimide lithium salt (1.70 g, 5.92 mmol, 6.23 eq., M = 287.09 g·mol^−1^) was stirred in CH_3_CN (6 mL) during 24 h at 40 °C. Then, the solvent was removed under vacuum. The crude product was washed with demineralized water to afford a pure product as light yellow solid (m = 0.60 g, n = 0.78 mmol, 81% yield). ^1^H NMR (400 MHz, DMSO-d_6_): 2.37 (s, 3H), 7.37 (d, 2H, ^3^*J* = 8.2Hz), 7.56 (d, 2H, ^3^*J* = 8.5 Hz) 7.90 (t, 2H, ^3^*J* = 7.8 Hz), 8.19 (d, 2H, ^3^*J* = 8.2 Hz), 8.34 (d, 2H, ^3^*J* = 8.5 Hz), 8.47–8.53 (m, 4H). ^19^F NMR (DMSO-d_6_) δ(ppm) −78.70 (s). ^13^C NMR (101 MHz, DMSO-d_6_) 21.6 (CH_3_), 113.2 (Cq), 116.4 (Cq), 120.1 (q, CF_3_, ^1^J_C-F_ = 320 Hz) 122, 6 (Cq, 2C), 128.1 (CH, 2C), 128.4 (Cq), 131.9 (CH, 2C), 132,1 (Cq), 133.4 (CH, 2C), 133.4 (CH, 2C), 135.7 (CH, 2C), 136.0 (CH, 2C), 136.6 (CH, 2C), 140.0 (Cq), 144.0 (Cq), 164.5 (C=O, 2C). HRMS (ESI MS) *m*/*z*: theor: 490.0299, found 490.0296 ((M-TFSI^−^)^+^ detected).

*(4-(1,3-Dioxo-1H-benzo[de]isoquinolin-2(3H)-yl)phenyl)(p-tolyl)iodonium hexafluoroantimonate (V)* (**6**): A mixture of (4-(1,3-dioxo-1*H*-benzo[*de*]isoquinolin-2(3*H*)-yl)phenyl)(*p*-tolyl)iodonium chloride (195.5 mg, 0.37 mmol, M = 527.77 g·mol^−1^) and sodium hexafluoroantimonate (V) (590.0 mg, 2.28 mmol, M = 258.74 g·mol^−1^) was refluxed in CH_3_CN (20 mL) for 7 days. After cooling, the solvent was removed under vacuum. The crude product was washed with water and filtered to afford a pure product as a light yellow solid (m = 0.14 g, 0.19 mmol, 51% yield). ^1^H NMR (400 MHz, Acetone-d_6_) δ 2.42 (s, 3H), 7.45 (d, 2H, ^3^*J* = 8.3 Hz), 7.69 (d, 2H, ^3^*J* = 8.6 Hz), 7.91 (t, 2H, ^3^*J* = 7.7 Hz), 8.29 (d, 2H, ^3^*J* = 8.3 Hz), 8.45–8.51 (m, 4H), 8.55 (d, 2H, ^3^*J* = 7.2 Hz). ^9^F NMR (Acetone-d_6_) δ −110.47–−136.42 (m, 6F). ^13^C NMR (DMSO-d_6_) δ(ppm): 20.9 (CH_3_), 113.4 (Cq), 116.5 (Cq), 122.4 (Cq, 2C), 127.3 (CH, 2C), 127.8 (Cq), 130.7 (CH), 130.8 (CH), 131.5 (Cq), 132.4 (CH, 2C), 132.6 (CH, 2C), 134.5 (CH), 134.7 (CH), 135.3 (CH, 2C), 135.6 (CH, 2C), 139.4 (Cq), 142.7 (Cq), 163.5 (C=O, 2C). HRMS (ESI MS) *m*/*z*: theor: 490.0299, found 490.0298 ((M-SbF_6_^−^)^+^ detected).

### 4.2. Other Materials

(3,4-Epoxycyclohexane)methyl-3,4-epoxycyclo-hexylcarboxylate (EPOX) and trimethylolpropane triacrylate (TMPTA) were selected as benchmark monomers for cationic and radical polymerization, respectively. These monomers were obtained from Allnex. The reference iodonium salt (Speedcure 938, Iod) was obtained from Lambson Ltd. (Wetherby, UK). Trimethylaniline (TMA) was purchased from Sigma-Aldrich (St. Louis, MO, USA). Their corresponding molecular structures are shown in Scheme 2.

### 4.3. Photopolymerization Reactions Monitored by Real Time Fourier Transformed Infrared Spectroscopy (RT-FTIR)

The weight percent of the photoinitiator is calculated from the monomer content (*w*/*w*). The photosensitive thin formulations (~25 µm of thickness) were deposited on a BaF_2_ pellet under air for the CP of EPOX. The 1.4 mm thick samples of EPOX were also polymerized under air into a rounded plastic mold of ~7 mm diameter and 1.4 mm of thickness. For thin and thick samples, the evolution of the epoxy group content of EPOX was continuously followed by real time FTIR spectroscopy (JASCO FTIR 4100, Tokyo Japan) at about 790 and 3700 cm^−1^, respectively. The procedure used to monitor the photopolymerization profiles has been described in detail in refs [65,66].

### 4.4. UV-Visible Absorption Spectroscopy

The UV-Visible absorbance properties of the different iodoniums were studied by JASCO V730 UV–visible spectrometer (JASCO, Tokyo Japan). To demonstrate the formation of acid upon irradiation, methyl red was added as pH indicator as reported in [67].

### 4.5. Electron Spin Resonance (ESR) Spin Trapping (ESR-ST)

Electron spin resonance-spin trapping experiments were carried out using an X-band spectrometer (Magnettech MS400, Magnettech, Berlin, Germany). The radicals were observed under nitrogen saturated media at room temperature. *N*-*tert*-Butylnitrone (PBN) was used as a spin trap agent in acetonitrile [66]. ESR spectra simulations were carried out using PEST WINSIM software.

### 4.6. Laser Write Experiments

A computer programmed laser diode with spot size around 50 μm was utilized for spatially controlled irradiation to produce specific 3D patterns using **5**/TMA in TMPTA monomer. Finally, the printed patterns were analyzed through a numerical optical microscope (DSX-HRSU, OLYMPUS corporation, Tokyo, Japan) [66].

### 4.7. Molecular Modeling

Frontier Molecular Orbital calculations were performed by the Gaussian 03 suite of programs (Pittsburgh, PA, USA) [68,69]. The simulation of UV absorption spectra and the calculations of the C–I bond dissociation energies (BDE) were done at the density functional theory level (UB3LYP/LANL2DZ) on the relaxed geometries calculated at this level.

## 5. Conclusions

In the present paper, the design and the synthesis of a new series of five iodonium salts combining within a unique chemical structure the naphthalimide chromophore and the iodonium scaffold are presented. Compared to the reference structure Speedcure 938, the proposed iodoniums are more reactive at 365 nm to initiate the cationic polymerization of epoxides. *Bis*(trifluoromethane)sulfonimide was found as the most interesting counter anion, the best photoinitiating abilities in both radical and cationic polymerizations being determined for the iodonium salt possessing this counteranion. The development of new 3D printing resins @405 nm based on the cationic polymerization of epoxides and based on the elaboration of a CTC between an iodonium salt and an amine has allowed to polymerize at longer wavelength than what is theoretically possible on the simple basis of the absorption spectrum of the iodonium salt. The formation of CTC to red-shift the absorption spectrum of a photoinitiator and to allow the polymerization at longer wavelength is an efficient tool that deserves to be further investigated. Especially, by mean of this innovative approach, benchmark UV photoinitiators that do not exhibit an absorption in the visible range could be revisited in the context of this recent approach.
